# Pressurized Liquid Extraction of Coumarins from Fruits of *Heracleum leskowii *with Application of Solvents with Different Polarity under Increasing Temperature

**DOI:** 10.3390/molecules17044133

**Published:** 2012-04-05

**Authors:** Krystyna Skalicka-Woźniak, Kazimierz Głowniak

**Affiliations:** Department of Pharmacognosy with Medicinal Plant Unit, Medical University in Lublin, 1 Chodzki, Lublin 20-093, Poland; Email: kglowniak@pharmacognosy.org

**Keywords:** Pressurized Liquid Extraction, coumarins, furanocoumarins, *Heracleum leskowii*

## Abstract

Coumarins are nowadays an important group of organic compounds from natural sources that are useful in a number of fields. Because they possess different pharmacological properties, finding the proper extraction conditions for their separation from plant matrices is a very important step. In this report Pressurized Liquid Extraction (PLE) under different temperature conditions and with different types of extraction solvents were tested. As a matrix, fruits of *Heracleum leskowii* have been used. A simple reverse phase high-performance liquid chromatographic method (RP-HPLC) coupled with a photodiode array detector (DAD) has been developed for separation and quantitative analysis of the main coumarins. Umbelliferone, xanthotoxin, angelicin, isopimpinellin, bergapten, imperatorin and isoimperatorin were investigated. Bergapten and imperatorin were dominant in almost all extracts in the range of 9.92 ± 0.02–20.93 ± 0.06 and 12.19 ± 0.98–19.07 ± 0.03 mg/100 g, respectively. Dichloromethane and methanol were chosen as the most proper suitable solvents for extraction of coumarins. By increasing the temperature the amount of extracted coumarins increases in petroleum ether and dichloromethane extracts.

## 1. Introduction

One of the major goals for the analytical chemist is to transfer as many of the analyte molecules of interest possible from the matrix without interfering substances [1,2]. One of the most frequently used liquid-solid extraction methods is Soxhlet extraction, developed in the late 19th century, but still used in many laboratories. Soxhlet extraction has been the leading technique used for a long time and still is considered to be a standard technique and the main reference to which all other new extraction methods are compared [3]. This method requires large volumes of organic solvents and long extraction times are needed. Additionally slow analyte diffusion and desorption from the sample matrix to the extraction fluid are characteristic. Soxhlet extractions also generate dirty extracts, so a long clean-up process is necessary [4]. 

One of the most popular techniques nowadays is Pressurized Liquid Extraction (PLE), also known as Accelerated Solvent Extraction (ASE). The technique is today well-established and has been used for the extraction of a great variety of compounds from numerous matrices [1]. Compared to classic extraction in a Soxhlet apparatus, complete PLE can be achieved in shorter time with a small volume of organic solvent and much better penetration of sample by the solvent [3]. Extraction parameters are thoroughly investigated in order to produce an exhaustive methodology, but exhaustiveness very often leads to co-extraction of unwanted, interfering matrix components, thus the selection of the proper solvent which must be able to solubilise the analyte and minimize co-extraction of other matrix components, is very important.

The aim of our study was to find suitable parameters for the extraction of furanocoumarins. Plants from *Heracleum* genus are known as possessing a wide range of coumarins of different polarities, thus fruits of *Heracleum leskowii* have been used as a plant matrix [5,6].

For extraction of pharmacologically active coumarins several methods have been applied, as well as different extraction solvents. Some previous experiments showed that the best single solvent for extraction of furanocoumarin from *Archangelica officinalis* fruits is petroleum ether. Addition of small amounts of organic modifiers, e.g., petroleum ether +30% dichloromethane, allowed decreasing the extraction time [7]. Fruits of *Heracleum candicans* were extracted in room temperature with methanol and aqueous solutions of methanol. Heraclenol was found to be maximum in 30% aqueous methanolic extract, while the maximum concentration of bergapten was found in the pure methanolic extract [8]. The extraction of eight coumarins of biological interest was carried out by Waksmundzka *et al*. [9]. Initially, petroleum ether, usually used in selective extraction of the furanocoumarin fraction from plant tissues, was employed, whereas the more polar coumarins were extracted with methanol. PLE, in most cases, was the most suitable for extraction of furanocoumarins from *Pastinaca sativa *fruits, compared with the other tested methods [9]. Similar results were achieved for fruits of *Archangelica officinalis* [10]. 

Coumarins are nowadays an important group of organic compounds from natural sources that are useful in a number of fields. Because they possess different pharmacological properties, finding proper extraction conditions for their separation from plant matrix is a very important step. As the PLE method was find as one of the most proper methods for extraction of coumarins, the aim of this study was finding the optimal parameters.

## 2. Results and Discussion

**Figure 1:**
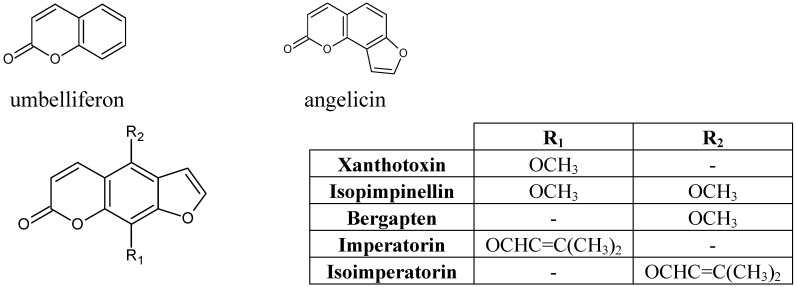
Chemical structures of identified compounds.

**Figure 2:**
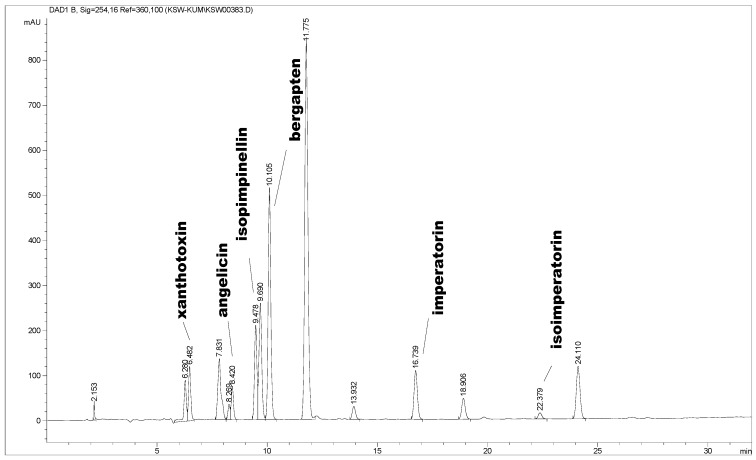
HPLC chromatogram of an extract of the fruits of *H. leskowii* obtained by PLE at 110 °C with dichloromethane as a solvent.

For qualitative and quantitative purposes, the chromatographic conditions were evaluated by taking into account the selectivity, linearity, accuracy and repeatability. No interfering peaks were observed in the blank chromatograms at the quantification wavelengths. The peak purity and degree of match with the standard spectra were greater than 98%. Good linearity (*R^2^* > 0.9994) was obtained for all the compounds within the ranges tested. Recoveries for fortified samples and for standards were in the range of 95.7–99.0% and 95.2–98.1%, respectively, with RSD not higher than 6%. The repeatability within-day and between days of peak areas, expressed by means of the percentage of relative standard deviation (%RSD), were lower than 5%. Under the applied chromatographic conditions, 7 coumarins were appropriately separated over a running time of 30 min. Additionally, no interfering peaks were detected. Structures of identified compounds are presented in Figure 1. Typical HPLC chromatogram is presented in Figure 2. The results from calibration together with recoveries are summarized in Table 1.

**Table 1:** Calibration curves, determination coefficient *R^2^* and recoveries for identified compounds with ± RSD (%).

Identified compound	Regression equation	*R^2^*	Recovery of fortified samples [%]	Recovery of standards [%]
Umbelliferon	y = 5380.9x − 19.533	0.9994	96.5 ± 5.9	95.2 ± 3.3
Xanothoxin	y = 2433.1x + 5.8727	0.9998	97.4 ± 5.8	96.2 ± 4.3
Angelicin	y = 2147.8x + 51.551	0.9994	96.8 ± 4.9	97.6 ± 4.9
Isopimpinellin	y = 2147.9x + 48.555	0.9995	95.7 ± 4.2	96.5 ± 3.6
Bergapten	y = 3671.6x + 8.9942	0.9999	98.0 ± 3.8	96.4 ± 6.0
Imperatorin	y = 1453.6x + 31.2	0.9994	99.0 ± 6.0	98.1 ± 4.1
Isoimperatorin	y = 2842.6x − 1.6606	0.9995	98.2 ± 5.7	98.0 ±5.1

### 2.1. Extraction Solvents

The extraction must be performed with the most adequate solvent and under ideally predetermined analytical conditions of temperature [11]. The extraction solvent must be able to solubilize the analytes of interest and minimize the co-extraction of other matrix components. When choosing the extraction solvent, it is also important to take into account the volatility of the solvent if extract concentration is necessary. The polarity of the solvent should be close to that of the target compounds [12].

**Figure 3:**
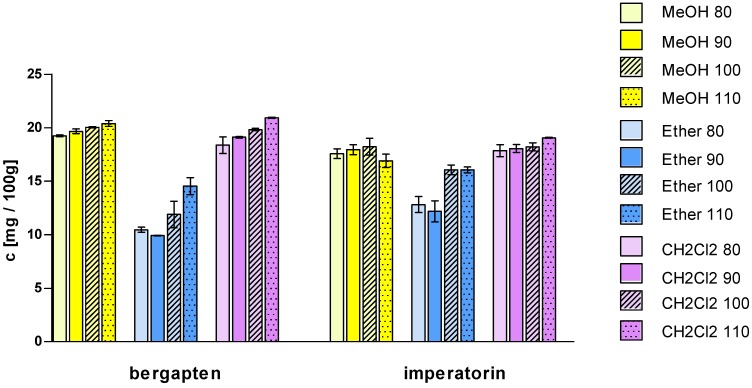
Extracted amounts of major coumarins in *H. leskowii* fruits under different extraction conditions.

As can be seen in Table 2 and Figure 3 the highest extraction yield of umbelliferon, angelicin and xanthotoxin was achieved when methanol was used as the extraction solvent. Methanol was slightly better for extraction of bergapten (the predominant compound) and isopimpinellin than dichloromethane, however the highest efficiency was achieved when dichloromethane was used at the temperature of 110 °C (20.93 ± 0.06 mg/100 g and 13.29 ± 0.37 mg/100 g, respectively). Imperatorin and isoimperatorin were also efficiently extracted by methanol but the proportion of those compounds in dichloromethane extracts was slightly higher. Petroleum ether, which was the most popular for extraction of furanocoumarins, showed the lowest extraction efficiencies. The hydroxycoumarin umbelliferon was not detected in nonpolar etheric extracts.

**Table 2:** Content of identified coumarins in the examined extracts obtained from fruits of *H. leskowii* (each value is the mean c = mg/100 g dry weight DW, n = 3), SD–standard deviation; RSD–relative standard deviation (%).

		Umbelliferon	Xanthotoxin	Angelicin	Isopimpinellin	Bergapten	Imperatorin	Isoimperatorin
**MeOH 80**	**C**	**1.85**	**16.32**	**7.11**	**12.68**	**19.25**	**17.58**	**1.31**
	**SD**	0.06	0.66	0.32	0.80	0.09	0.45	0.02
**MeOH 90**	**C**	**1.75**	**15.28**	**5.80**	**13.16**	**19.67**	**17.96**	**1.32**
	**SD**	0.04	0.40	0.07	0.07	0.21	0.46	0.02
**MeOH 100**	**C**	**1.80**	**12.60**	**5.77**	**13.01**	**20.04**	**18.24**	**1.34**
	**SD**	0.04	0.24	0.04	0.59	0.06	0.78	0.01
**MeOH 110**	**C**	**1.78**	**11.50**	**5.70**	**12.72**	**20.39**	**16.91**	**1.37**
	**SD**	0.01	0.87	0.02	1.27	0.27	0.63	0.01
**Ether 80**	**C**		**5.86**	**2.88**	**6.44**	**10.47**	**12.82**	**0.89**
	**SD**	Nd	0.16	0.05	0.35	0.24	0.75	0.07
**Ether 90**	**C**		**4.30**	**2.86**	**6.15**	**9.92**	**12.19**	**0.97**
	**SD**	Nd	0.35	0.29	0.24	0.02	0.98	0.09
**Ether 100**	**C**		**5.68**	**3.48**	**7.91**	**11.90**	**16.06**	**1.16**
	**SD**	Nd	0.39	0.66	1.40	1.23	0.46	0.09
**Ether 110**	**C**		**8.48**	**4.07**	**9.42**	**14.55**	**16.07**	**1.24**
	**SD**	Nd	0.04	0.12	0.77	0.80	0.28	0.01
**CH2Cl2 80**	**C**	**0.90**	**13.64**	**5.17**	**12.19**	**18.37**	**17.87**	**1.33**
	**SD**	0.01	0.07	0.12	0.43	0.77	0.55	0.01
**CH2Cl2 90**	**C**	**1.06**	**13.84**	**5.29**	**12.38**	**19.12**	**18.06**	**1.33**
	**SD**	0.01	0.15	0.04	0.32	0.07	0.37	0.01
**CH2Cl2 100**	**C**	**1.24**	**14.24**	**5.42**	**11.99**	**19.83**	**18.22**	**1.36**
	**SD**	0.01	0.22	0.13	0.42	0.11	0.38	0.02
**CH2Cl2 110**	**C**	**1.33**	**14.35**	**5.61**	**13.29**	**20.93**	**19.07**	**1.39**
	**SD**	0.07	0.06	0.12	0.37	0.06	0.03	0.01

Obtained results showed that no significance differences were observed in the extraction yield. For isopimpinellin, bergapten, imperatorin and isoimperatorin, dichloromethane was only slightly better. Therefore methanol can be applied, without detriment in the extraction yield, instead of the toxic and flammable dichloromethane, which has been extensively used for the extraction of coumarins

### 2.2. Extraction Temperature

Temperature is a very important parameter in PLE, which adds to the often observed increased recoveries compared to other extraction techniques. However some compounds are temperature sensitive and can be degraded [1]. Too low temperatures may cause decrease in the extraction efficiency. In fact 100 °C is the most commonly applied temperature overall. This is most likely caused by a good balance between good extraction efficiency with relatively moderate co-extraction of undesired matrix components [1].

Increasing the temperature from 80 to 110 °C increased the extracted amount of target compounds, what is easily seen when dichloromethane and petroleum ether are used as extractant solvents. Extraction with dichloromethane at temperature 110 °C took effect in highest recovery of isopimpinellin, bergapten, imperatorin and isoimperatorin.

Isolation of more polar compounds like umbelliferone, xanthotoxin and angelicin with methanol is more effective at lower temperature, so those constituents are more susceptible to degradation. The highest yield of those compounds was achieved after extraction of methanol at temperature 80 °C (1.85 ± 0.06 mg/100 g, 16.32 ± 0.66 mg/100 g and 7.11 ± 0.32 mg/100 g, respectively).

Previous experiments of Waksmundzka-Hajnos *et al*. [10] showed that by increasing the temperature from 100 to 130 °C, the amount of umbelliferone and xanthotoxin extracted increased. For bergapten and isopimpinellin the increase in the extraction yield was moderate, whereas imperatorin and phellopterin showed a decreasing tendency. In our experiments 110 °C was the maximum temperature used. When methanol was used as the extraction solvent, a decrease of the extracted amount of umbelliferone, xanthotoxin and angelicin was observed. Similarly, like in experiments cited above, for bergapten the increase in the extraction yield was moderate and the extraction yield of imperatorin at the highest temperature was lower. Together with increasing the temperature, the amount of extracted coumarins increases in petroleum ether and dichloromethane extracts. It can be explained with increasing the ability of the solvent to solubilize the compounds and decreasing the viscosity of liquid solvent, allowing better penetration of the extractant into the matrix.

### 2.3. Combination with SPE

When choosing the extraction solvent, it is also important to take into account the compatibility with the later treatment steps, especially extract clean-up [12]. Application of the proper solvent gives more selective extraction but always unwanted matrix components occurs in extracts. Combining the extraction under proper conditions with Solid-Phase Extraction improves the removal of interfering substances, separation of the coumarin fraction and sample concentration.

## 3. Experimental

### 3.1. Plant Material

Fruits of *Heracleum leskowii* L. (Umbelliferae) were collected in the Medicinal Plant Garden, Department of Pharmacognosy, Medical University in Lublin, Poland, in Summer 2009. The plant material was identified by Mrs Krystyna Dąbrowska, Botanical Garden of Maria Curie-Skłodowska University in Lublin, a specialist in botany. The fruits were dried at room temperature and ground to a powder. Voucher specimen No 26/27-28 is deposited in the herbarium of The Department of Pharmacognosy, Medical University, Lublin, Poland.

### 3.2. Solvents and Standards

All solvents used for extraction were purchased from The Polish Reagents (POCh, Gliwice, Poland). Methanol used for HPLC was of chromatographic grade (J.T. Baker Inc., Deventer, The Netherlands). Water was purified using a Simplicity^TM^ system (Millipore, Molsheim, France). Standards of coumarins were purchased from Carl Roth GmbH (Karlsruhe, Germany). 

### 3.3. Pressurized Liquid Extraction

Pressurized Liquid Extraction was performed with a Dionex ASE 100 instrument (Dionex, Sunnyvale, CA, USA). The plant material (exactly weighted 1 g) was placed into a 10 mL stainless steel extraction cell. In order to optimize extraction parameters four different temperatures were tested: 80, 90, 100 and 110 °C with extraction solvents of different polarity: dichloromethane, petroleum ether and methanol. The static time of extraction process was 10 min. After the extraction process, the extraction cell content was flushed using the same extractant in the amount equal to 60% of the extraction cell volume. The obtained extracts were evaporated to dryness, dissolved in methanol and transferred into 10 mL calibration flasks. All procedure was repeated three times.

### 3.4. Solid Phase Extraction

In order to isolate the coumarin fractions from other components, a previously established SPE method [13] was applied. Octadecyl BakerBond SPE-microcolumns (500 mg, 3 mL, J.T. Baker, Phillipsburg, NJ, USA) were activated with methanol (10 mL), followed by water (10 mL) and 80% aqueous methanol solution (10 mL). After that 80% aqueous solutions of each extract (10 mL) were filtered through the columns under reduced pressure (SPE-12G chamber, J.T. Baker, Grossgerau, Germany). Eluates were collected to 10 mL volumetric flasks.

### 3.5. High-Performance Liquid Chromatography

The content of coumarins in different extracts obtained from fruits of *Heracleum leskowii* were determined by HPLC, performed with an Agilent 1100 system (Agilent Technologies, Palo Alto, CA, USA) coupled with an auto-sampler, a column thermostat and a DAD detector. The 250 mm × 4.6 mm stainless steel column, packed with 5 μm Hypersil BDS C_18_ (Shandon, Cheshire, UK) was used. The flow rate was 1 mL/min, the column temperature was 25 °C. A stepwise mobile phase gradient was prepared from methanol (A) and water (B). The gradient was: 0-5 min 50–60% A; 5–25 min 60–80% A; 25–30 min isocratic 80% A; 30–40 min 80–100% A. Peak identification was achieved by comparison of both the retention time and UV absorption spectrum with those obtained for individual standards. The quantitative determination was performed using the following wavelengths: λ = 254, 280 and 320 nm [14]. 

### 3.6. Method Validation

The proposed analytical method was carefully evaluated in terms of selectivity, linearity, accuracy and repeatability. The selectivity of the method was evaluated by comparing the chromatograms of the extracts of representative samples to those of the method blank (extraction solvent) and to a solution of standards. Peak shapes, retention times, and spectral purity of the chromatographic peak were considered in order to detect possible interferences. The linearity was checked by means of the external standard method. Thus, each calibration curve was analyzed three times with five different concentrations: 0.1; 0.075; 0.05; 0.025; 0.01 mg/mL. The calibration curves were characterized by their regression coefficient, slope of the line (b) and intercept of the straight line with y-axis (a). In order to establish the linear ranges, the determination coefficients (*R^2^*) were considered. Measurement of intra-day and inter-day variability was used to determine the repeatability of the method. The intra-day repeatability was examined on six individual samples within one day, and inter-day repeatability was determined on three different days. The accuracy of the SPE method was evaluated through recovery studies for fortified samples and for standards. The mixtures of the standards at the known concentration were passed through the SPE column. Purification of the sample was performed according to described method. Additionally methanolic solution of standards at three concentration levels were prepared and added to the extract containing the known amount of target compounds, then extracts were purified using SPE method. The recovery data were obtained from the relationship between the amount of standard added and the amount detected.

## 4. Conclusions

Combination of small-scale PLE method together with SPE followed by a HPLC method is a convenient procedure by which the concentrations of umbelliferone, xanthotoxin, angelicin, isopimpinellin, bergapten, imperatorin and isoimperatorin, the important active components in *H. leskowii *fruits, can be isolated and monitored. The effect of the most important parameters in PLE, *i.e.*, extraction temperature and solvent, were evaluated. Dichloromethane and methanol were chosen as the most suitable solvents for the extraction of coumarins. By increasing the temperature, the amount of extracted coumarins increases in petroleum ether and dichloromethane extracts. Bergapten and imperatorin were dominant in almost all extracts
